# Bone marrow mesenchymal stem cell-derived exosomes promote plasminogen activator inhibitor 1 expression in vascular cells in the local microenvironment during rabbit osteonecrosis of the femoral head

**DOI:** 10.1186/s13287-020-01991-2

**Published:** 2020-11-11

**Authors:** Lu Li, Yikai Wang, Xiaobing Yu, Yongming Bao, Lijia An, Xiaowei Wei, Weiting Yu, Baoyi Liu, Junlei Li, Jiahui Yang, Yan Xia, Ge Liu, Fang Cao, Xiuzhi Zhang, Dewei Zhao

**Affiliations:** 1 National-Local Joint Engineering Laboratory for the Development of Orthopedic Implant Materials, Affiliated Zhongshan Hospital of Dalian University, Dalian, Liaoning People’s Republic of China; 2 Department of Orthopedics, Affiliated Zhongshan Hospital of Dalian University, Dalian, Liaoning People’s Republic of China; 3 Medical College of Dalian University, Dalian, Liaoning People’s Republic of China; 4 School of Bioengineering, Dalian University of Technology, Dalian, Liaoning People’s Republic of China; 5 Department of Pathology, Affiliated Zhongshan Hospital of Dalian University, Dalian, Liaoning People’s Republic of China

**Keywords:** Nontraumatic osteonecrosis of the femoral head, PAI-1, Bone marrow mesenchymal stem cells, Exosome, miRNA

## Abstract

**Background:**

Nontraumatic osteonecrosis of the femoral head (NONFH) is a highly disabling orthopedic disease in young individuals. Plasminogen activator inhibitor 1 (PAI-1) has been reported to be positively associated with NONFH. We aimed to investigate the dysregulating PAI-1 in bone marrow mesenchymal stem cells (BMMSCs) and vascular cells in rabbit steroid-induced NONFH.

**Methods:**

To verify the hypothesis that BMMSCs could promote thrombus formation in a paracrine manner, we collected exosomes from glucocorticoid-treated BMMSCs (GB-Exo) to determine their regulatory effects on vascular cells. microRNA sequencing was conducted to find potential regulators in GB-Exo. Utilizing gain-of-function and knockdown approaches, we testified the regulatory effect of microRNA in exosomes.

**Results:**

The expression of PAI-1 was significantly increased in the local microenvironment of the femoral head in the ONFH model. GB-Exo promoted PAI-1 expression in vascular smooth muscle cells and vascular endothelial cells. We also revealed that miR-451-5p in GB-Exo plays a crucial role for the elevated PAI-1. Moreover, we identified miR-133b-3p and tested its role as a potential inhibitor of PAI-1.

**Conclusions:**

This study provided considerable evidence for BMMSC exosomal miR-mediated upregulation of the fibrinolytic regulator PAI-1 in vascular cells. The disruption of coagulation and low fibrinolysis in the femoral head will eventually lead to a disturbance in the microcirculation of NONFH. We believe that our findings could be of great significance for guiding clinical trials in the future.

**Supplementary Information:**

The online version contains supplementary material available at 10.1186/s13287-020-01991-2.

## Background

Nontraumatic osteonecrosis of the femoral head (NONFH or nontraumatic-ONFH) is a chronic and destructive disease in orthopedics with high disability rates. Glucocorticoid (GC) usage is one of the main risk factors [1]. GC-induced NONFH is assumed to cause thrombosis to occur in bone blood vessels, leading to a sequential process of blood flow obstruction, venous high pressure, arterial ischemic, osseous hypoxia, loss of bone integrity, and collapse of the subchondral bone [2, 3]. Plasminogen activator inhibitor 1 (PAI-1) can suppress the conversion of plasminogen to plasmin and reduce the levels of fibrinolysis in blood vessels, leading to the formation of thrombus [4, 5]. Numerous studies have established a strong association between abnormal PAI-1 levels or activity and a wide range of diseases. Most PAI-1-related human diseases are caused by elevated antigenic concentrations and their inhibitory activity, which are associated with venous thrombosis [4, 6, 7].

Accumulating evidence has indicated the associations between NONFH and PAI-1, and most of them have been found to be a consequence of increased or aberrant PAI-1 in patients with NONFH [8, 9]. The most reasonable pathomechanism is that increased PAI-1 reduces fibrinolytic function, affects microcirculation in the femoral head, leads to femoral head blood flow reduction, and eventually causes ischemic ONFH [10, 11]. Upregulated PAI-1 was only identified in the serum of patients with NONFH, but not in osteoarthritis, rheumatoid arthritis, and fracture patients, and so it has the potential to be a diagnostic marker for NONFH [12]. Although steroid treatment can promote plasma PAI-1 levels in the entire body or in a specific type of cells, there are few systematic and conclusive studies about the mechanism of PAI-1 regulation disorder in the femoral head microenvironment of NONFH.

In recent years, stem cell treatments, such as the use of bone marrow mesenchymal stem cells (BMMSCs) to treat ONFH, have achieved good results in both animal models and clinical trials [13, 14]. However, the basic mechanism of BMMSC transplantation treatment remains incompletely understood. Exosomes have gradually attracted attention because they can function in cell-to-cell signaling and can release internal regulatory factors from BMMSCs into the extracellular space, thereby influencing processes in surrounding recipient cells [15]. Growing evidence supports the ideas that exosomes have an important role in different diseases and physiological changes [16, 17]. At present, recent studies have shown that exosomes mediate regenerative functions in ONFH and other ischemic diseases [18–20]. It has been reported that the release and function of exosomes can be regulated and controlled by glucocorticoids [21]. Moreover, Al-Nedawi et al. demonstrated that exosomes from perivascular cells, such as mast cells, have a significant activating effect on PAI-1 expression in endothelial cells [22]. However, in the pathomechanism of steroid-induced ONFH, whether BMMSC-derived exosomes can interfere with the local blood circulation of the femoral head has not been reported.

Selective removal of some miRNAs in exosomes has also been suggested to be a rapid way of regulating gene expression during the progression of ONFH [23]. Most miRNAs have only been analyzed intracellularly, and few investigations have focused on the role of miRNAs in the extracellular environment. BMMSC miRNAs that are secreted into the extracellular environment could act as key modulators for surrounding tissue cells. Thus, the role of miRNA-mediated cross-talk between BMMSCs and vascular tissues in steroid-induced ONFH remains elusive.

In our previous work, we have successfully constructed a well-validated model of steroid-induced ONFH in rabbit [24], and we verified the preventive effect of ONFH with a combination of an anticoagulant and a vasodilator in systemic administration [25]. But we still have no idea about local microcirculation disorder in the femoral head. Consequently, in this study, we hypothesized that BMMSC-derived exosomes mediate impaired fibrinolytic activity in the early stage of ONFH, and upregulated PAI-1 induce thrombosis in the local femoral head vascular microenvironment. The purpose of this study was to unveil the role of PAI-1 in GC-induced ischemic ONFH in vitro and in vivo. We also revealed that exosomes derived from BMMSCs could be secreted into the extracellular environment to regulate PAI-1 expression in vascular cells. Gene sequencing analysis would provide us with clues for exploring the pathomechanism and identifying potential solutions for hypofibrinolysis in ONFH.

## Materials and methods

### Animals

All animal care and experimental procedures were approved by the Ethical Committee for Animal Research of Affiliated Zhongshan Hospital of Dalian University. Male 3-month-old healthy adult New Zealand white rabbits weighing 3–4 kg were purchased from Dalian Medical University, Liaoning, China. The rabbits were kept in specialized animal centers at the Affiliated Zhongshan Hospital of Dalian University and were acclimated for 4 days before initiation of studies.

### Animal model and grouping

NONFH rabbit models were generated by previously well-validated protocols [26]. All rabbits were first randomly divided into 2 groups. The rabbits in the experimental (GC) group (*n* = 8) were intravenously injected with 10 μg/kg lipopolysaccharide (Sigma, USA). After 24 h, the experimental (GC) group was intramuscularly injected with 20 mg/kg of methylprednisolone acetate (Pfizer Manufacturing Belgium, USA) 3 times every 24 h: the control group (*n* = 8) and the normal group, which was given the same volumes of saline. miR-451-5p group (*n* = 3): random three of the control group rabbits received AgomiR-451-5p (25 nmol, Genepharma Co., Lt., China) at a dose of 1 mL by intravenous injection one week after first injection of saline. miR-133b-3p group (*n* = 3): random three of the experimental (GC) group rabbits received agomiR-133b-3p (25 nmol, Genepharma Co., Lt., China) at a dose of 1 mL by intravenous injection 1week after the first injection of methylprednisolone acetate during modeling procedure. All rabbits were sacrificed 4 weeks later, and the organs and bone tissue of the lower extremities of the rabbits in the four groups were obtained. For protein extraction, tissue samples were homogenized in liquid nitrogen and then dissolved in ice-cold protein buffer. For RNA isolation, tissue samples were snap-frozen in liquid nitrogen.

### Cell isolation and culture

BMMSCs were isolated from the femoral head of healthy rabbits by bone marrow aspiration as described [27]. The cells were cultured in Dulbecco’s modified Eagle’s medium/Ham’s F12 nutrient medium (DME/F12, Hyclone, GE, USA) with 10% fetal bovine serum (FBS, HyClone; GE, USA) and 1% antibiotic antimycotic solution and changed every 2 days. Vascular endothelial cells (VECs) and vascular smooth muscle cells (VSMCs) were isolated from rabbit thoracic aortas by digestion with different enzymes as described [28, 29]. VECs were maintained in endothelial cell medium (ECM, ScienCell Research Laboratories, USA) and cultured in culture flasks that were coated with gelatin, and the medium was changed every 1–2 days. VSMCs were cultured in the same medium as what was used for BMMSCs, and the medium was changed every day. The cells were separately identified by flow cytometry and immunohistochemistry. All cells were incubated at 37 °C with 5% CO_2_. Cells from passages 3–5 were used in experiments.

### Characterization of rabbit cells

BMMSCs and VECs were identified by flow cytometry. Details on the experimental procedures are provided in Supplementary material. Characterization of VSMCs was performed by alpha-smooth muscle actin (α-SMA)-labeled VSMC immunohistochemistry analysis. Detailed procedures are also provided in the supplementary material.

### Micro-CT assay

Micro-CT (Siemens, Inveon Micro-CT, Berlin, Germany) images were obtained to evaluate trabecular bone structure of the femoral head. The scanning protocol was 80 kV and 500 μA, with an effective pixel size of 15.48 μm. Based on the CT images, a volume of interest (VOI) was selected from these regions for three-dimensional reconstruction and values of bone volume/total volume (BV/TV), bone surface area/bone volume, trabecular thickness (Tb.Th, mm), and trabecular spacing (Tb.Sp, mm) were compared.

### Histological and immunohistochemistry (IHC) analysis

The expression of PAI-1 was observed by IHC analysis. Paraffin sections were dewaxed by routine methods, and antigens were retrieved. After that, 10% bovine serum albumin (BSA, HyClone; GE, USA) was incubated with the slides in a humid box at room temperature for 60 min, and then primary rabbit anti-PAI-1 antibody (1:500; Abcam, USA) was incubated with the slides overnight at 4 °C. After washing three times in PBS, the sections were incubated with poly-HRP goat anti-rabbit IgG at 37 °C for 30 min. Then, the sections were stained with a DAB dye solution and restained with hematoxylin. In addition, hematoxylin and eosin (H&E) staining and Masson’s trichrome staining were conducted to evaluate the histological morphology. The sections were observed under an optical microscope. The number of empty lacunae in five random regions in each section (5 sections in each group) was counted, and the percentage of empty lacunae was defined as the ratio of empty lacuna number to the total lacuna count.

### Alkaline phosphatase (ALP) staining

After 3 days of glucocorticoid stimulation, cell culture medium was removed; then, cells were determined by ALP kits (Beyotime Biotechnology, China) according to the manufacturer’s user guide. All cells were observed under a microscope.

### Oil red O staining

After 3 days of glucocorticoid stimulation, cell culture medium was removed. Cells were washed with PBS and fixed with 4% paraformaldehyde solution at room temperature for 30 min. Then, the freshly prepared Oil Red O working solution (Sangon Biotech, China) was added into culture flask and incubated with the cells at room temperature for 60 min. The staining solution was removed and cells were washed with PBS 3 times. All cells were observed under a microscope.

### Isolation and purification of exosome

BMMSCs were seeded at 5 × 10^5^ cells per 25 cm^2^ culture flask in 5 mL of culture medium and were reached confluence 5 days later. The cells were cultured in medium supplemented with 10% serum without FBS-derived exosomes and methylprednisolone (0 or 5 μg/mL). Over the next 72 h, the culture medium was collected. The exosomes were collected from the 40 mL of medium and were subsequently purified via an exosome concentration kit (Liaoning Rengen Biosciences, China) according to the manufacturer’s information. The samples were stored at − 80 °C.

### Co-culturing BMMSC exosomes with vascular cells

VECs and VSMCs were seeded at a density of 10^5^ cells/well in a 24-well plate in a 1-mL culture medium before co-culture treatment. After overnight plating, cells were treated with BMMSCs (GC treated or not) exosome for 72 h. The same dose of exosomes (20 μL) was added to each group.

### Nanoparticle tracking analysis (NTA)

Particle sizes of exosomes were measured by dynamic light scattering (DLS) (Zetaview, Particle Metrix, Germany).

### Transmission electron microscope (TEM)

Exosomes were dropped on a copper grid for 3 min. Excess liquid was removed with filter paper and dried at room temperature. Next, 3% ammonium molybdate negative staining solution (Solarbio Science & Technology, China) was added to the copper grid for 3 min. After removing excess staining solution with filter paper, the grid was examined, and TEM images were recorded (JEM-2100, JEOL, Japan).

### Enzyme-linked immunosorbent assay (ELISA)

The concentration of PAI-1 protein was determined by rabbit PAI-1 ELISA kit (Shanghai Lengton Biotechnology, China) according to the manufacturer’s instructions.

### Quantitative real-time polymerase chain reaction (qRT-PCR)

All cells were lysed with RNAiso Plus (Takara Biomedical Technology, Japan) and reverse transcribed using a PrimeScript™ RT reagent kit with gDNA Eraser (Takara Biomedical Technology, Japan) following the manufacturer’s instructions. The generated cDNA was amplified using TB Green™ Premix Ex Taq™II (Takara Biomedical Technology, Japan). The primers (SERPINE1, ocu-miR-133b-3p, ocu-miR-451-5p, U6, and GAPDH) (see Table S1 in the online-only supplementary material) were synthesized by TAKARA BIO (Takara Biomedical Technology, Japan). Target mRNA expression levels were normalized to GAPDH, using the 2^-△△Ct^ method, and the analysis results are presented as fold change.

### Western blotting

Western blotting was conducted as described [30]. Details on the western blotting experimental procedures are provided in supplementary material. All the antibodies mentioned were as follows: PAI-1 (1:1000) (OmnimAbs, USA), GAPDH (1:1000) (Novusbio, USA).

### MicroRNA sequencing

Extracting and purifying total RNA from cell supernatants was performed via SeraMir™ exosome RNA amplification kit (System Biosciences, USA). The purified total RNA was analyzed via Novogene’s genomics platform via illumina HiSeq™2500/MiSeq for gene clustering and sequencing. Other detailed procedures are provided in the supplementary material.

### Transfection of miR-133b-3p mimics and inhibitor

miR-133b-3p mimics (AgomiR), inhibitor (AntagomiR), stable negative control, and inhibitor N.C. were synthesized by Genepharma Co., Lt. (China) (Table S2). They were transfected into vascular endothelial cells at a final concentration of 40 nM using lipofectamine 2000 (Invitrogen, USA) according to the manufacturer’s instructions. All cells were collected for qRT-PCR and western blotting 48 h after transfection.

### Statistical analyses

Every experiment was repeated at least three times. All data was analyzed by using SPSS version 23.0 (IBM Corp., Armonk, USA). Measurement data were expressed as the mean ± standard error of mean (s.e.m). Comparisons between groups were analyzed by *t* tests or ANOVA. A *P* value of < 0.05 was considered statistically significant.

## Results

### Elevation of PAI-1 expression in GC-induced rabbit ONFH in vivo

**Fig. 1:**
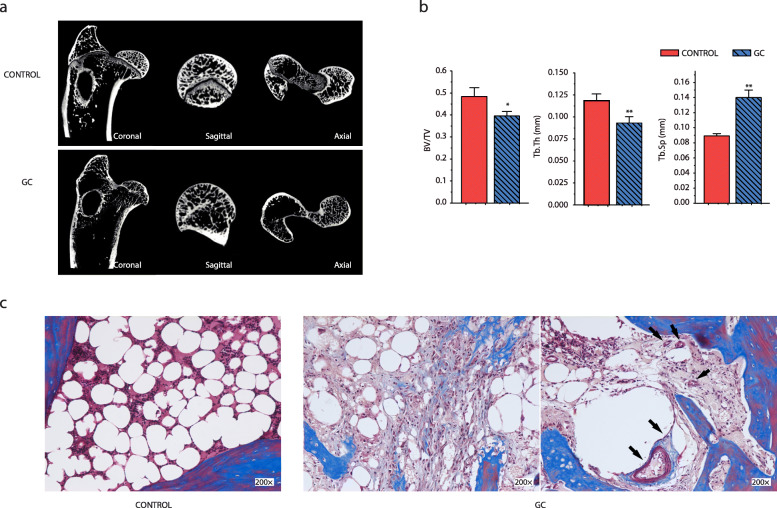
Representative micro-CT and Masson staining images of GC-induced rabbit ONFH. **a** Reconstructed coronal, sagittal, and axial images of femoral head within the control and experimental (GC) groups. In the control group, the trabecular bone showed a better structural integrity, whereas in the experimental (GC) group, the trabecular bone was sparse and relatively thin. **b** Quantitative CT analysis. Quantitative analysis showed that BV/TV, Tb.Th were all significantly lower in the experimental (GC) group compared to that of the control group. Asterisks (*) indicate significant differences (*p* < 0.05) and **p < 0.01. The results are presented as the means ± s.e.m. **c** Histological morphology of the bone marrow in GC-induced ONFH by Masson’s trichrome staining. The bone marrow was replaced by degenerated hypertrophic adipocytes and necrotic tissues in the experimental (GC) group. Hyperplasic and thickened small vessels endothelium where also found, as well as empty lacunae in the GC group. Magnification: ×200. Black arrows indicate the intraosseous vessels

Firstly, the experimental (GC) group rabbits (*n* = 5) all showed typical signs of early stage of ONFH. Micro-CT results demonstrated the trabecular bone appeared thinner and sparser. The trabecular space was larger in the subchondral bone in the experimental (GC) group (Fig. 1a). Compared with control group, the mean rate value of BV/TV and Tb.Th in experimental group were all significantly lower (*p* < 0.05 and *p* < 0.01), whereas the Tb.Sp was significantly higher (*p* < 0.01) (Fig. 1b). On the histologic evaluation, clear differences were observed between the two groups. From Fig. 1c, it can be clearly seen that hypertrophic adipocytes had degenerated accompanied by hyperplasia, even becoming necrotic in the experimental group. Thrombophilia/hypofibrinolysis state was noticed in the Masson staining images. After treatment with glucocorticoid and endotoxin, perivascular fibrosis and neointima formation can be found in the experimental group, as well as hyperplasic and thickened small vessel endothelium. Moreover, most bone lacunae are empty. The micro-CT and staining results indicated the success of the rabbit ONFH models.

**Fig. 2:**
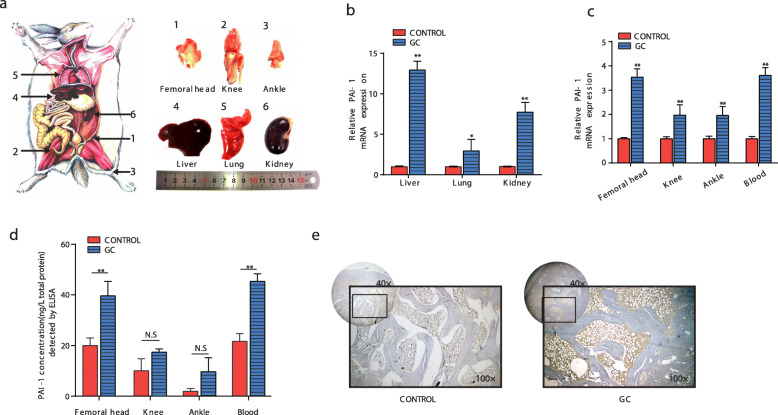
Elevation of PAI-1 in GC-induced rabbit ONFH in vivo. **a** Organ and bone tissue of the lower extremities in rabbit were tested (*n* = 5). **b** Expression of PAI-1 of organs was significantly increased following GC treatment, as shown by qRT-PCR. **c** Bone tissue from the lower extremities and blood samples presented a significant increase of PAI-1 expression following GC treatment, as shown by qRT-PCR. **d** PAI-1 concentrations in the femoral head, knee, ankle, and blood samples were remarkably increased after the GC treatment, as shown by ELISA. **e** Representative immunohistochemical staining of PAI-1 in samples of the femoral head from the control and GC-induced experimental groups. Magnification: ×40. Enlarged images shown in the lower right corner at ×100. Asterisks (*) indicate significant differences (*p* < 0.05) and **p < 0.01. The results are presented as the means ± s.e.m

To investigate PAI-1 expression in major organ tissues and weight-bearing joints in the lower limbs of GC-induced ONFH rabbit models, we extracted and examined the following tissue samples: major organs (liver, kidney, and lung), hip joint bone (femoral head), knee joint bone (distal femur, proximal tibia, and fibula), ankle joint bone (distal tibia and fibula, talus), and peripheral blood samples (Fig. 2a). First, the expression of PAI-1 mRNA in all experimental groups was significantly higher than it was in the normal control groups (*p* < 0.01). There was a greater increase in the mRNA expression in the liver than there was in the other organs (Fig. 2b). In joint bone tissues, the PAI-1 expression in the femoral head of the experimental group exhibited the greatest increase, which was very similar to that of the blood samples. It is worth noting that the relative mRNA expression in the femoral head region was markedly higher than it was in the other joints (Fig. 2c). Moreover, with a fixed amount of total protein, we found that the PAI-1 concentration in the femoral head and blood were significantly higher than that of the corresponding normal control group (*p* < 0.01). However, we noticed that before and after modeling, the PAI-1 concentrations in the ankle and knee groups showed no significant difference (*p* > 0.05) due to their inherent low abundance (Fig. 2d). Further verifying the change of PAI-1 expression in the femoral head region, the immunohistochemical results showed that the PAI-1 in the local microenvironment of the femoral head in the experimental group was notably higher than it was in the normal control group (Fig. 2e).

### Characteristics of BMMSCs under GC stimulation and the expression of PAI-1 in vitro

**Fig. 3:**
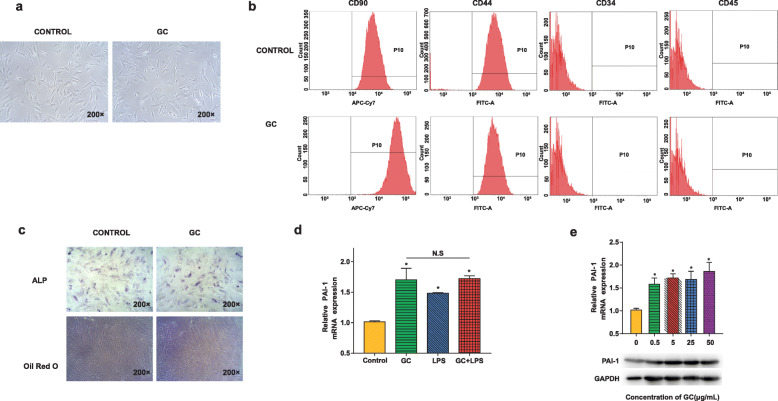
Characterization of BMMSCs and significantly upregulated PAI-1 affected by GC treatment in vitro. **a** Representative BMMSCs exhibited a typical spindle-shaped morphology with or without GC-treatment. Magnification: ×200. **b** Flow cytometry analysis of the BMMSC cell surface markers (positive for CD90 and CD44 and negative for CD34 and CD45). **c** Representative alkaline phosphatase staining (top) and oil red O staining (bottom) of BMMSCs from the control and GC-treated groups. Magnification: ×200. **d** Expression of PAI-1 in BMMSCs was significantly increased following treatment with GC, lipopolysaccharide, and a combination of the two, as shown by qRT-PCR. **e** Expression of PAI-1 in BMMSCs was significantly increased following treatment with any concentration of GC, as shown by qRT-PCR and western blotting. Asterisks (*) indicate significant differences (*p* < 0.05 by ANOVA). The results are presented as the means ± s.e.m

BMMSCs were isolated from the femoral bone marrow cavity of healthy adult rabbits. Figure 3a and b show visualization of spindle-shaped BMMSCs adherently cultured and BMMSC-specific cell surface antigens (positive for CD90 and CD44 and negative for CD45 and CD34). However, after BMMSCs were stimulated with 5 μg/mL methylprednisolone for 3 days, the morphological differences were not observed between the experimental group and the normal control. We did not find changes in surface antigenic features after GC stimulation (Fig. 3b). Moreover, there were no increased numbers of lipid droplets found in GC-treated BMMSCs by oil red O staining. The ALP staining results also showed that there was no obvious difference between the two groups. These results indicated that BMMSCs did not display a tendency of differentiation into adipocytes or osteoblasts, and they maintained their BMMSC characteristics at our prescribed methylprednisolone concentration and duration time (Fig. 3c). To illustrate the effect of combined methylprednisolone and endotoxin treatment on PAI-1 expression in BMMSCs, the in vivo doses were calculated and adopted for in vitro experiments. Then, 5 μg/mL methylprednisolone, 100 ng/mL endotoxin, or both combined doses were added into BMMSC culture medium. PAI-1 expression in BMMSCs was significantly upregulated in all three groups after the drug treatments (*p* < 0.05), but there was no significant difference among the three groups, indicating that there was no synergistic effect between the two drugs (Fig. 3d). Both qRT-PCR and western blotting results displayed the same trend of significantly increased PAI-1 expression (*p* < 0.05) as glucocorticoid concentration increased. However, no statistical differences were found among concentration gradients (Fig. 3e).

### Characterization of exosomes from glucocorticoid-stimulated BMMSCs

**Fig. 4:**
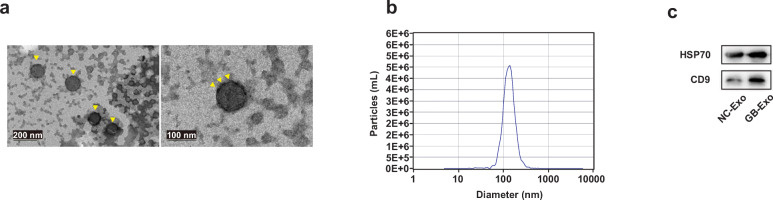
Characterization of exosomes derived from BMMSCs. **a** Morphology of exosomes examined by TEM. Arrow heads indicate apparent exosome. Scale bars, 100 nm and 200 nm. **b** Particle size distribution of exosomes measured by DLS. **c** Western blotting analysis of the surface biomarkers CD9 and heat shock proteins HSP70

Since BMMSCs did not show a differentiation trend, we tried to determine whether they affected other cells with their paracrine exosomes. To fully characterize rabbit BMMSC-derived exosomes, we used exosome-free serum to culture BMMSCs for 3 days under different conditions. First, NTA showed that the particle size distribution of the isolated vesicles was between 30 and 150 nm, and more than 99% of the microvesicles had a particle size of 130 nm (Fig. 4a), which is consistent with previous reports of exosomal particle size distribution. TEM provided us with visual evidence that the typical circular membrane-bound vesicle structure with a size of approximately 100 nm (Fig. 4b). Western blotting results confirmed the exosomal characteristics by analysis of surface antigen markers; signals were positive for CD9 and HSP70 in the model and control groups (Fig. 4c). For subsequent experiments, we then collected exosomes derived from GC-treated and untreated normal BMMSCs.

### BMMSC exosomes regulated PAI-1 expression in VECs and VMSCs

**Fig. 5:**
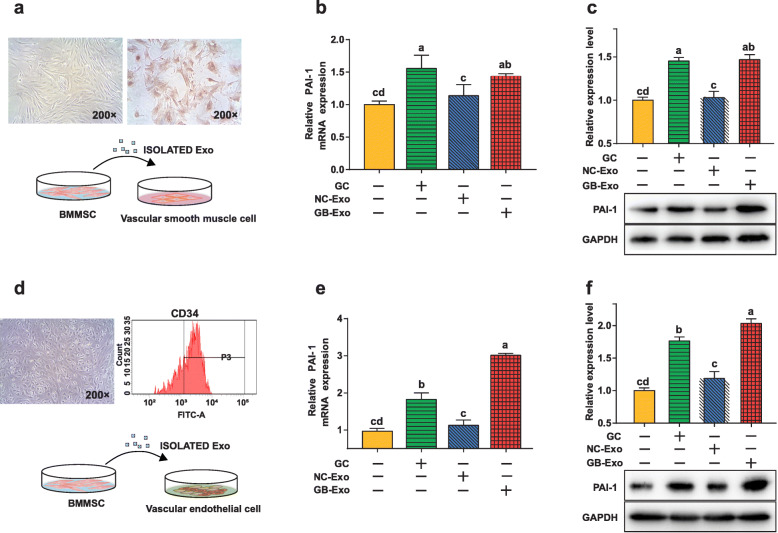
Exosomes derived from BMMSCs with GC treatment activated VSMCs and VECs to secrete PAI-1. **a** Representative VSMCs exhibited a typical spindle-shaped morphology (top left). Representative α-SMA immunohistochemical staining of VSMCs (top right). Magnification: ×200. Schematic diagram of the experimental design (bottom). **b** Expression of PAI-1 in VSMCs was increased by GC treatment and by GB-Exo treatment, as shown by qRT-PCR and **c** western blotting results. **d** Representative VEC morphology (top left). Flow cytometry analysis of the cell surface markers on VECs (positive for CD34) (top right). Schematic diagram of the experimental design (bottom). **e** qRT-PCR and **f** western blotting also demonstrated that overexpressed PAI-1 was detected in GC-treated and GB-Exo groups in VECs. In particular, GB-Exo groups exhibited dramatically upregulated PAI-1 expression in VECs. Values in each column with different letters present significant differences (*p* < 0.05 by ANOVA). The results are presented as the means ± s.e.m

To explore whether BMMSCs could induce intravascular fibrinolytic disorders through paracrine effects after GC stimulation and whether BMMSC exosomes could regulate the expression of PAI-1, we conducted a series of experiments in primary VMSCs and VECs from New Zealand white rabbits. As shown in Fig. 5a, isolated rabbit primary VMSCs were identified by their morphology under a microscope (×200) and by the α-SMA-labeled immunohistochemical results. In the schematic diagram (Fig. 5a), GC-treated BMMSC exosomes (GB-Exo) and normal control BMMSC exosomes (NC-Exo) were cocultured with VMSCs. First, we found that PAI-1 expression in VMSCs was significantly upregulated by GC treatment as demonstrated in BMMSCs. NC-Exo did not significantly promote the expression of PAI-1 in VMSCs. However, as we assumed, GB-Exo significantly upregulated PAI-1 expression, which was in contrast to the result of the control group. Moreover, there was no significant difference between the GB-Exo group and the GC-treated group (Fig. 5b, c).

Primary rabbit VECs were identified based on the results of morphological microscopic examination and flow cytometry results (positive for CD34) (Fig. 5d). Similar to the above experimental design for VECs, after GC treatment, VECs also upregulated PAI-1 expression, and NC-Exo still did not increase PAI-1 expression. Intriguingly, we found that PAI-1 expression in the GB-Exo group was significantly higher than it was in the control group and GC-treated group (Fig. 5e and f), which means BMMSCs have a stronger effect on the expression of PAI-1 in VECs than what was achieved following the direct stimulation by GC in vitro. These data suggested that exosomes of BMMSCs have a notable effect on the promotion of PAI-1 in vascular cells.

### Exosomal miRNA from BMMSCs regulates PAI-1 expression in VECs

**Fig. 6:**
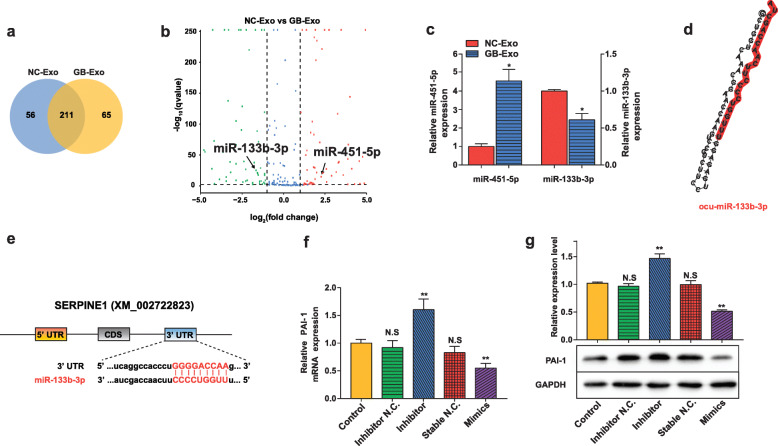
Exosomal microRNAs from BMMSCs regulated PAI-1 expression in vascular cells. **a** Venn diagram illustrating the overlap of DEGs by miRNA sequencing. **b** Volcano plot of global miRNA changes with specks denoting genes with significant fold changes (green specks for downregulated miRNAs and red specks for upregulated miRNAs). **c** A significant increase of miR-451-5p expression and a significant reduction of miR-133b-3p expression in GB-Exo groups were detected by qRT-PCR. **d** Predicted secondary structure of the miR-133b-3p precursor. The whole sequence is the precursor of miRNAs, and the red prominent part is the mature body sequence. **e** The binding sites for miR-133b-3p within the PAI-1 gene sequence (SERPINE1). **f** qRT-PCR results demonstrated that the expression levels of PAI-1 were reduced by treatment with miR-133b-3p mimics and increased by treatment with an miR-133b-3p inhibitor in VECs, which was also verified by western blotting (**g**). Asterisks (*) indicate significant differences (*p* < 0.05) and **p < 0.01. The results are presented as the means ± s.e.m

To understand the properties of BMMSC exosomes for their regulation of PAI-1 in VECs and VMSCs, we examined the content of GB-Exos. Since microRNAs were recently shown to be the main regulators enriched in exosomes that were important for cell-cell communication, we performed microRNA sequencing from GB-Exos and NC-Exos. Differentially expressed genes sequencing (DEGseq) analysis provided some clues as to the dysregulated miRNAs induced by GC treatment. The DEGseq results showed that 332 exosomal miRNAs were significantly differentially expressed between the two groups, of which 211 were coexpressed miRNAs, 56 were expressed only in NC-Exos, and 65 were expressed only in GB-Exos (Fig. 6a). In a volcano plot of global miRNA expression (Fig. 6b), an overexpressed microRNA (miR-451) was identified in the right cluster. Relative expression of miR-451-5p in exosomes was also confirmed by qRT-PCR (Fig. 6c). We believe that miR-451 might be responsible for the elevated PAI-1 expression in vascular cells. miR-451 has been reported to suppress the PI3K/AKT pathway in various cells [31, 32], and the PI3K/AKT pathway can negatively regulate the expression of PAI-1 [33, 34], resulting in miR-451 indirectly promoting PAI-1 expression.

To find a miRNA that can directly bind to PAI-1 to induce its downregulation, we screened multiple miRNAs that matched the PAI-1 gene (SERPINE1) in the sequencing results. Excitingly, miR-133b-3p was the only miRNA that was negatively correlated with the expression of target genes. miR-133-3p was significantly downregulated in GB-Exos (Fig. 6b and c). The secondary structure of the miR-133b-3p precursor is shown in Fig. 6d. Using the microRNA target scanning algorithm, we predicted the potential binding site of miR-133b-3p to the target gene (mRNA-XM_002722823 encoded by SERPINE1) (Fig. 6e). To further determine whether miR-133b-3p could downregulate PAI-1 levels, miR-133b-3p inhibitors (antagomiR), mimics (agomiR), and negative controls (miR-NC) were transfected into VECs. Our qRT-PCR and western blotting results showed that PAI-1 was significantly downregulated in the miR-133b-3p mimics group. In contrast, the inhibitors increased PAI-1 expression in vitro (Fig. 6f and g). Thus, these results supported the idea that miR-133b-3p have the potential to restore PAI-1 dysregulation caused by GC treatment.

### Effects of miR-451-5p and miR-133b-3p on GC-induced ONFH in vivo

**Fig. 7:**
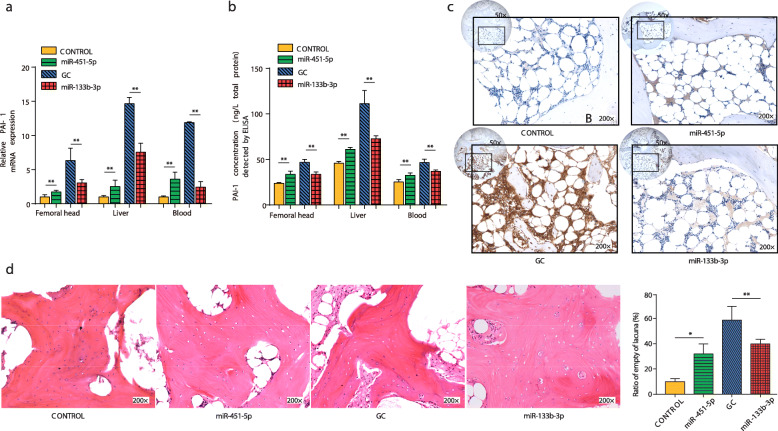
Functional influence of exosomal miRNAs on regulating PAI-1 expression in GC-induced rabbit ONFH models. **a** Expression of PAI-1 from the femoral head bone, liver, and peripheral blood samples by qRT-PCR. **b** PAI-1 concentrations in the femoral head bone, liver, and peripheral blood samples by ELISA. AgomiR-451-5p significantly increased PAI-1 expression in all tested samples. AgomiR-133b-3p significantly decreased PAI-1 expression from GC treatment. **c** Representative IHC images of PAI-1 staining of the harvested femoral head tissues in different treatment. PAI-1 is expressed in a dark brown color. Magnification: ×50. Enlarged images shown in the lower right corner at ×200. **d** Representative HE staining images of subchondral bone from femoral head and semi-quantitative analysis. The miR-451-5p group showed significantly more empty lacunae than that in the control group, and compared with the GC group, the miR-133b-3p group showed significantly fewer empty lacunae. Magnification: ×200. Asterisks (*) indicate significant differences (*p* < 0.05) and **p < 0.01. The results are presented as the means ± s.e.m

In order to testify the impact of miR-451-5p and miR-133b-3p on the rabbit model of GC-induced ONFH in vivo, we evaluated the pattern of PAI-1 expression from liver, femoral head bone, and peripheral blood samples by qRT-PCR (Fig. 7a) and ELISA (Fig. 7b). Firstly, the results demonstrated that miR-451-5p could directly upregulated PAI-1 expression in vivo, the relative expression of PAI-1 in miR-451-5p group revealed significantly higher levels compared to the control group in all tested tissue samples (*p* < 0.01) (Fig. 7a and b).

Meanwhile, to investigate the preventive effects of miR-133b-3p on GC-induced ONFH, the rabbit model of ONFH was induced by intramuscularly injection of glucocorticoid, followed by intravenously administration of AgomiR 133b-3p or an equal volume saline. As exhibited in Fig. 7a and b, miR-133b-3p exerted the therapeutic function of downregulating the disorganized PAI-1 overexpression. The results showed that PAI-1 expression in miR-133b-3p group was dramatically decreased than that in the GC group (*p* < 0.01).

In histological examinations, the femoral head IHC results (Fig. 7c) indicated that the miR-451-5p group possessed much more PAI-1 positive areas than the control group, and the PAI-1 negative area of the miR-133b-3p group was clearly larger than that of the GC group. These trends were in accordance with the molecular test results. To further evaluate the indirect effects of miR-451-5p and miR-133b-3p on ONFH progression, the trabecular lacunae measured by HE staining analysis. The apoptosis of osteocytes led to the appearance of more empty lacunae. In Fig. 7d, it can be observed the most of lacunae were filled with osteocytes in the control group. However, there were much more empty lacunae and obviously condensed nuclei in osteocytes were found in miR-451-5p group. Additionally, compared with the GC group, the percentage of empty lacunae had decreased significantly (*p* < 0.01) with AgomiR 133b-3p injection. These results confirmed our in vitro hypothesis, they indicated that inhibition of miR-451 and miR-133 can remarkably affect the progression of GC-induced ONFH by regulating PAI-1 expression level in local microenvironment.

## Discussion

Hypofibrinolysis (reduced ability to lyse thrombi) mediated by PAI-1 has been reported as a considerable pathogenic factor in NONFH. PAI-1 can combine with and inactivate tissue-type plasminogen activator (TPA) and urokinase-type plasminogen activator (UPA), and inhibit the conversion of plasminogen to plasmin, which can degrade fibrin in thrombosis. Thrombophilia and hypofibrinolysis are the main risk factors in blood supply impediment, which induces impaired circulation to femoral head. Our current study presented exploratory in vivo evidence to support a previously unrecognized “idiopathic site” role suspected for PAI-1. We reasoned that local fibrinolytic dysfunction might explain why the hip joint is the region that is most frequently affected by osteonecrosis. Utilizing a widely used rabbit GC-induced ONFH model, our observation was that the local femoral head had the most prominent response to GC stimulation. Although GC leads to an increase in systemic PAI-1 levels, which is dissimilar from other weight-bearing joints, the exceptionally high PAI-1 expression in the femoral head directly leads to alternations in fibrinolysis in that local area. Furthermore, remarkable GC-induced increases in PAI-1 were demonstrated in a variety of rabbit primary-cultured cells in vitro, including BMMSCs, VSMCs, and VECs, which are the main component of the vascular structure in the femoral head. Our results further confirmed previous studies; as major fibrinolytic regulators, there is a close and vital association between PAI-1 dysregulation and GC-induced ONFH.

BMMSCs are a type of pluripotent cell. BMMSCs are particularly appealing for the treatment of ONFH. Several reports have found that BMMSC transplantation exhibited a reduced time to collapse, pain score improvements, and decreased lesion sizes with core decompressions [35, 36]. Here, this study revealed that in our in vitro experimental conditions, short-term GC treatment has not yet driven BMMSCs to differentiate into other types of daughter cells. The effect of BMMSCs on vascular structure and tissue cells was the prominent effect. Thus, BMMSCs have played a regulatory role in vascular cells through paracrine exosomes before differentiation occurs. In addition, the role of endotoxin was investigated in a rabbit ONFH model. We found that endotoxin and glucocorticoids have a similar impact on reducing fibrinolytic capacity and can significantly increase the expression of PAI-1, but the combination did not exhibit a synergistic effect.

Utilizing exosomes derived from different stem cells has recently become a popular area of research for treatment of ONFH [18, 19, 37]. Our previous research has revealed the widespread blood supply network of the femoral head [38]. This inspired current study on intravascular coagulation and hypofibrinolysis in the femoral head. First, it is observed that GC not only reduced the fibrinolytic capacity of BMMSCs and vascular cells, but also indirectly exacerbated the loss of fibrinolytic capacity in vascular cells via BMMSC-regulating effect. Notably, this indirect influence is even greater than what was observed with direct GC treatment in vitro, especially in VECs. The same pattern was found by Al-Nedawi and colleagues in mast cells [22]. This phenomenon also made us realize that BMMSCs could affect the entire femoral head by virtue of the widespread vascular network. In particular, in the early stage of ONFH with large doses of GC treatment, a thinner vein would be more susceptible to the effects we observed. In addition, venous elasticity is weaker. Once a vein thrombosis was formed, it was more likely to cause changes in venous blood flow, leading to early venous stasis in ONFH.

Recent studies have revealed that stem cells could regulate the expression of specific genes in peripheral tissue cells by exosome-mediated transportation of miRNAs [39]. In this work, we screened a large number of differentially expressed miRNAs by comprehensive deep sequencing analysis of the small RNA profile from exosomes released by BMMSCs. It should be noted that miR-451-5p was found in the overexpressed miRNAs in exosomes derived from GC-treated BMMSCs. Several studies have confirmed that overexpression of miR-451 inhibited the PI3K/AKT signaling pathway by downregulated CAB39 [31, 32]. Meanwhile, the PI3K/AKT pathway was also reported to negatively regulate PAI-1 expression in vascular endothelial cells through suppressed TNF-α [33]. Another study suggested that a reduction in active Akt results in elevated PAI-1 expression in cancer cells [34]. Collectively, we reasoned that the overexpression of miR-451 in exosomes plays a crucial role in eventually upregulation of PAI-1 levels in vascular cells. Our in vivo experiments also supported this conclusion. Finally, an exosomal miRNA (miR-133b-3p) from BMMSCs was discovered and its regulatory effect on PAI-1 was demonstrated. miR-133b-3p can target the 3′UTR of PAI-1 mRNA to reduce the expression of PAI-1. Based on our in vitro and in vivo results, we believe that engineered exosomal miR-133b-3p applied to the bone marrow of the femoral head might be a potentially efficient therapeutic approach for GC-induced ONFH.

## Conclusion

In summary, our results demonstrated characteristically elevated PAI-1 levels and a procoagulant state in GC-induced rabbit ONFH model. Significantly dysregulated PAI-1 in the femoral head region might explain the susceptibility to idiopathic ONFH. This study provided considerable evidence for BMMSC exosomal miR-mediated upregulation of the fibrinolytic regulators PAI-1 in vascular cells. Moreover, future mechanistic studies of exosome-mediated BMMSC modulatory secretion will provide important insights into how this paracrine pathway contributes to ONFH physiology and pathology. Our study provides a new perspective on the etiology of NONFH.

## Supplementary Information

**Additional file 1:** Supplementary data. Characterization of rabbit cells. MicroRNA sequencing. Western blotting. **Table S1.** Primers used for RTqPCR. **Table S2.** Synthesized miR-133b-3p mimics and inhibitors.

## Data Availability

All data generated and/or analyzed in this study are included in this published article (and its supplementary information files).

## Abbreviations

ONFH: Osteonecrosis of the femoral head
PAI-1: Plasminogen activator inhibitor 1
GC: Glucocorticoid
BMMSCs: Bone marrow mesenchymal stem cells
3’UTR: 3′-Untranslated region
GB-Exo: GC-treated BMMSC exosome
NC-Exo: Normal control BMMSC exosomes
ALP: Alkaline phosphatase
IHC: Immunohistochemistry
VECs: Vascular endothelial cells
VSMC: Vascular smooth muscle cells
TPA: Tissue-type plasminogen activator
UPA: Urokinase-type plasminogen activator
DEGseq: Differentially expressed gene sequencing
TEM: Transmission electron microscopy
NTA: Nanoparticle tracking analysis
DLS: Dynamic light scattering
ELISA: Enzyme-linked immunosorbent assay
qRT-PCR: Quantitative real-time polymerase chain reaction
