# Supplementary material for: Bone marrow mesenchymal stem cell-derived exosomes promote plasminogen activator inhibitor 1 expression in vascular cells in the local microenvironment during rabbit osteonecrosis of the femoral head
Source: Stem Cell Res Ther. 2020 Nov 11;11:480. doi: 10.1186/s13287-020-01991-2 (PMC7656701; doi:10.1186/s13287-020-01991-2)
Supplement: Supplementary file 1 — Additional file 1: Supplementary data. Characterization of rabbit cells. MicroRNA sequencing. Western blotting. Table S1. Primers used for RTqPCR. Table S2. Synthesized miR-133b-3p mimics and inhibitors. [file 13287_2020_1991_MOESM1_ESM.docx]

**Supplementary data**

***Characterization of rabbit cells***

BMMSCs cultured in the aforementioned medium were supplemented with methylprednisolone acetate (0, 5 μg/mL) for 72 h at 37°C. Then, the cells were washed with PBS and were removed by treatment with a 0.5% trypsin solution for 3 mins at 37°C, and they were centrifuged at 400x g for 10 mins at room temperature. The cells were washed three times, and anti-rabbit CD34, CD44 and CD45 (Biosynthesis Biotechnology, China), and CD90 antibodies (BioLegend, USA) were added to the cells. After incubation at 37°C for 15 min, the cells were analyzed using a flow cytometer (BD FACSD via software; version 7.0; BD Biosciences). VECs were identified by the same method using a CD34 antibody. Characterization of VSMCs was performed by alpha-smooth muscle actin (α-SMA) -labeled VSMCs immunohistochemistry analysis. VSMCs were placed in paraformaldehyde, and incubated at room temperature for 15 mins. After a PBS wash, the cells were washed again with PBS solution containing 0.4% Triton X-100 (Amresco, USA) and then were incubated at room temperature for 30 mins. Then, the cells were incubated at 4°C overnight with an anti-rabbit α-SMA antibody (1:1000, Biosynthesis Biotechnology, China). Next, the cells were incubated with a DAB detection kit (ZSGB-BIO, China) according to the manufacturer's information.

***MicroRNA sequencing***

Extracting and purifying total RNA from cell supernatants was performed via SeraMir^TM^ exosome RNA amplification kit (System Biosciences, USA). The purified total RNA was analyzed via Novogene’s genomics platform via illumina HiSeq^TM^2500/MiSeq for gene clustering and sequencing. Next, the small RNA tags were mapped to reference sequence by Bowtie without mismatch to analyze the RNA expression and distribution in relation to the reference. Mapped small RNA tags were used to identify known miRNAs. miRBase20.0 was used as a reference, and modified software mirdeep2 was used to identify potential miRNAs and draw secondary structures. The characteristics of the hairpin structure of miRNA precursors were used to predict novel miRNAs. The available software miREvo and mirdeep2 were integrated to predict novel miRNAs by exploring secondary structures.

***Western blotting***

All cells were seeded into 100 mm dishes and were cultured at 37°C with 5% CO2 for 3 days. Protease inhibitor and radio-immunoprecipitation assay lysate (Sigma Aldrich, Germany) were mixed with cells and used to extract total protein from cells. The total protein concentration was determined using a Bradford assay kit (Sangon Biotech, China). The samples were mixed with 4X loading buffer and were boiled at 98% for 4 mins. Then, 20 μL of each protein sample was separated by 10% gel via SDS-PAGE for 100 mins, and then was transferred to 0.22 μm polyvinylidene diﬂuoride (PVDF) membranes (EMD Millipore, Billicera, USA) at 300 mA for 70 min. Following blocking in 5% bovine serum albumin (Sigma Aldrich, Germany) with TBS Tween 20 (1xTBS, 0.1% Tween 20, PBS) for 60 mins at room temperature. The membranes were incubated overnight at 4°C with antibodies for PAI-1 (1:1000) (OmnimAbs, USA) and GAPDH (1:1000) (Novusbio, USA). Thereafter, the membranes were incubated with HRP-labeled anti-rabbit secondary antibodies (1:10000) for 1 h at room temperature and were visualized via enhanced electrochemiluminescence (ECL, Thermo Fisher Scientific, USA); they were then analyzed via a GIS gel image analysis system (Tanon Science and Technology, China).

**Table S1. Primers used for RT-qPCR.**

| Gene | Sequence |
| --- | --- |
| SERPINE1 | Forward:*5'*‑ ACGGTCAAGCAGGTGGACT ‑*3'* |
|  | Reverse: *5'*‑ GAGGGCATTCACCAGCAG ‑*3'* |
| GAPDH | Forward: *5'*‑ ATCACTGCCACCCAGAAGAC ‑*3'* |
|  | Reverse: *5'*‑ GTGAGTTTCCCGTTCAGCTC ‑*3'* |
| ocu-miR-133b-3p | Forward: *5’*-GTCCCCTTCAACCAGCTAAAA -*3’* |
| ocu-miR-451-5p | Forward: *5’*- CGCGAAACCGTTACCATTACTGAGTTT -*3’* |
| U6 | Forward:*5’*-GGAACGATACAGAGAAGATTAGC-*3’* |
|  | Reverse: *5’*-TGGAACGCTTCACGAATTTGCG -*3’* |

**Table S2. Synthesized miR-133b-3p mimics and inhibitors**

| Gene | Sequence (*5'* to *3'*) |
| --- | --- |
| Agomir | UUU GGU CCC CUU CAA CCA GCU A  GCU GGU UGA AGG GGA CCA AAU U |
| Antagomir | UAG CUG GUU GAA GGG GAC CAA A |
| Stable Negative Control | Sense UUC UCC GAA CGU GUC ACG UTT  Antisense ACG UGA CAC GUU CGG AGA ATT |
| Inhibitor N.C. | CAG UAC UUU UGU GUA GUA CAA |
